# *Gracilaria gracilis* (Gracilariales, Rhodophyta) from Dakhla (Southern Moroccan Atlantic Coast) as Source of Agar: Content, Chemical Characteristics, and Gelling Properties

**DOI:** 10.3390/md19120672

**Published:** 2021-11-26

**Authors:** Zahira Belattmania, Sanaa Bhaby, Amal Nadri, Khaoulaa Khaya, Fouad Bentiss, Charafeddine Jama, Abdeltif Reani, Vitor Vasconcelos, Brahim Sabour

**Affiliations:** 1 Phycology, Blue Biodiversity and Biotechnology RU, Laboratory of Plant Biotechnology, Ecology and Ecosystem Valorization—CNRST Labeled Research Unit N°10, Faculty of Sciences, University Chouaib Doukkali, P.O. Box 20, El Jadida 24000, Morocco; belattmania.z@ucd.ac.ma (Z.B.); sanaa.bhaby@gmail.com (S.B.); amal.nadri.ucd@gmail.com (A.N.); khaya.khaoulaa@gmail.com (K.K.); abreani@yahoo.fr (A.R.); sabour.b@ucd.ac.ma (B.S.); 2 Laboratory of Catalysis and Corrosion of Materials, Faculty of Sciences, University Chouaib Doukkali, P.O. Box 20, El Jadida 24000, Morocco; fbentiss@gmail.com; 3 Materials and Transformations Unit, University of Lille, CNRS, INRAE, Centrale Lille, UMR 8207-UMET, F-59000 Lille, France; Charafeddine.Jama@ensc-lille.fr; 4 CIIMAR, Interdisciplinary Centre of Marine and Environmental Research, University of Porto, Terminal de Cruzeiros do Porto de Leixões, Av. General Norton de Matos, s/n, 4450-208 Matosinhos, Portugal; 5 Department of Biology, Faculty of Sciences, University of Porto, Rua do Campo Alegre, 4169-007 Porto, Portugal

**Keywords:** *Gracilaria gracilis*, agar quality, spectroscopic characterization, gel properties

## Abstract

Agar is a sulfated polysaccharide extracted from certain marine red algae, and its gel properties depend on the seaweed source and extraction conditions. In the present study, the seaweed *Gracilaria gracilis* (Gracilariales, Rhodophyta) from Dakhla (Moroccan Atlantic Coast) was investigated for its agar content, structure, and gel properties. The agar yields of *G. gracilis* were 20.5% and 15.6% from alkaline pretreatment and native extraction, respectively. Agar with alkaline pretreatment showed a better gelling property supported by higher gel strength (377 g·cm^−2^), gelling (35.4 °C), and melting (82.1 °C) temperatures with a notable increase in 3,6-anhydro-galactose (11.85%) and decrease in sulphate (0.32%) contents. The sulfate falling subsequent to alkaline pretreatment was verified through FT-IR spectroscopy. The ^13^C NMR spectroscopy showed that alkaline-pretreated agar has a typical unsubstituted agar pattern. However, native agar had a partially methylated agarose structure. Overall, this study suggested the possibility of the exploitation of *G. gracilis* to produce a fine-quality agar. Yet, further investigation may need to determine the seasonal variability of this biopolymer according to the life cycle of *G. gracilis*.

## 1. Introduction

**Figure 1:**
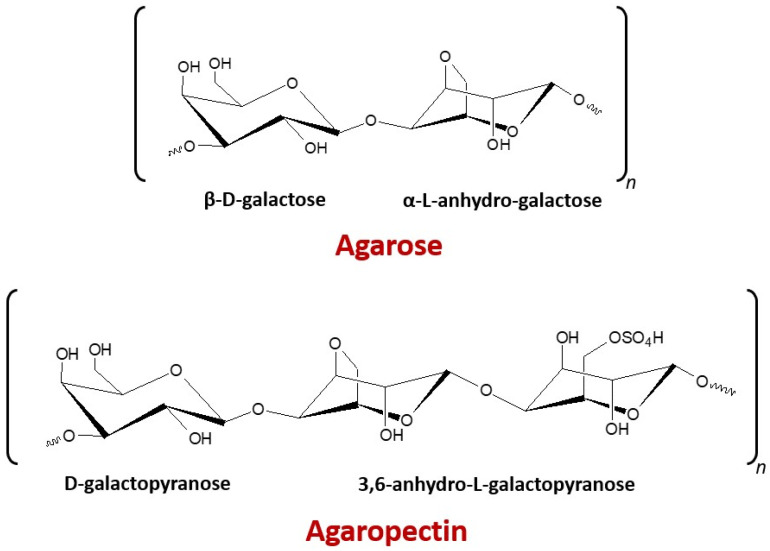
Chemical structures of repeating saccharide units of agar.

Seaweed’s cell wall and other components in the cellular matrix are made up of mostly structural polysaccharides existing as a heteropolysaccharide complex. A large amount of these polymers is sulfated, which includes the agar (with two polysaccharides mixtures, namely agarose and agaropectin; Figure 1) extracted from marine red algae known as agarophytes [1]. Agar applications are mainly based on their gel characteristics. More than 80% of the agar produced is consumed by the food industry and the rest is utilized mainly in biotechnology and other scientific applications [2]. *Gracilaria* and *Gelidium* species are mainly used for commercially producing agar. *Gracilaria* is preferred for the production of food-grade agar, whereas *Gelidium* is used for the production of pharmaceutical-grade agar and agarose [3,4]. Moreover, agar from *Gelidium* is better quality with an interesting gel strength, gelling, and melting temperatures [5,6], while a low-quality agar gel is obtained from *Gracilaria* spp. related to its high sulfate content [7]. However, the gelling properties of agars from *Gracilaria* species can be enhanced by an alkali pretreatment to convert α-l-galactose-6-sulfate into 3,6-anhydro-α-l-galactose [8].

Over the years, due to the growing demand for agar, natural stocks of agarophytes have been overexploited leading to a deficiency of wild raw material for agar production [9]. In 2015, the global agar market was estimated to be USD 214.98 million, which is further anticipated to grow at 4.9% CAGR from 2016 to 2025 [10]. Accordingly, the culture of agar-producing seaweeds, especially *Gracilaria* spp., is initiated in several countries [11]. The quantity of farmed *Gracilaria* species rose from 933,000 tons in 2005 [12] to 3.6 million tons in 2019, contributed to by 11 countries, including six countries in Eastern and South-Eastern Asia, two countries in South America (Chile and Brasil), two countries in Northern Africa (Morocco and Tunisa), and one European country (Spain) [13]. In fact, the culture of *Gracilaria* could enhance its production in other regions to respond to agar market demand [14]. During the last decade, Morocco has adopted a new development and competitiveness strategy for the aquaculture sector. The Halieutis plan that identified the development of aquaculture as a growth driver of the fishery sector created the national agency for the development of aquaculture. One of the priority actions of this Agency was the identification of suitable areas for aquaculture development. Currently, suitable natural sites for *Gracilaria* farming are recognized to supplement the natural resources for agar production. In addition, the increasing demand for seaweed products and the need for fishermen to seek alternative or additional livelihoods resulted in the emergence of seaweed farms in some locations along the Moroccan coastlines such as Marchika lagoon and Dakhla bay [15], with 273 tons of Moroccan farmed *Gracilaria* actually supplied to the global seaweed market [13]. *Gracilaria gracilis* (Gracilariales, Rhodophyta) is among the agarophytes species proposed to be most farmed in Dakhla bay. Unfortunately, the data regarding the content and quality of the agar from this species are not available. In this context, the present work aims to investigate the physicochemical characterization of agar from *G. gracilis* collected on natural deposits at Dakhla (South of Morocco), which constitutes the main cutting source of *G. gracilis* farming projects.

## 2. Results and Discussion

### 2.1. Agar Content

The agar yields of *G. gracilis* are shown in Table 1. The native extraction had a yield of 15.16 ± 2.5% dw. Agar yield increased to 20.5 ± 1.3% dw when alkaline treatment (6% NaOH) was used prior to extraction. According to Yarnpakdee et al. [16], alkaline pretreatment can destabilize the cross-links occurring in the cell wall of *Gracilaria* species. This allowed the release of more agars from a swollen or disrupted structure during extraction. Fidelis et al. [17] reported that native extraction (using water only) of sulphated polysaccharides from *Gracilaria birdae* had a lower yield, compared to the combination method of an alkaline solution, proteolysis, and sonication. Similarly, Praiboon et al. [18] highlighted that the agars extracted from *Gracilaria fisheri* and *G. edulis* using alkaline pretreatment (5% NaOH) have a higher yield compared to native extraction. Nevertheless, alkaline pretreatment at temperatures exceeding 80 °C caused a decrease in the yield of agar due to the degradation of polysaccharides and agar loss associated with diffusion during the pretreatment process [19,20,21,22].

### 2.2. Structural Characterization

#### 2.2.1. FT-IR Analysis

**Figure 2:**
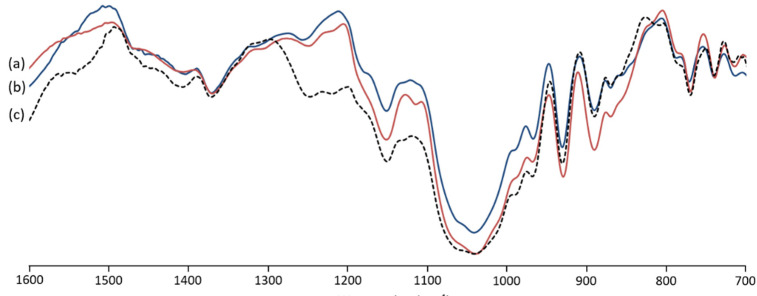
FT-IR spectra of commercial agar (**a**) compared to alkaline pretreated (**b**) and native (**c**) agars from *Gracilara gracilis*.

The FT-IR spectra of *G. gracilis* extracted agar showed significant similarity with the analyzed commercial agar (Figure 2). The typical bands of agar were, commonly, situated at 700–1400 cm^−1^ [23]. The band located at 738 cm^−1^ is attributed to C-O-C binding [24]. The band at 805 cm^−1^ recorded in native extracted agar (Figure 2c) is recognized as sulfate groups at C-2 of 3,6 anydro-l-galactose [23,24]. The weak band at 855 cm^−1^ is attributed to the sulfate groups at C-4 of D-galactose [25,26,27]. The band located at 886 cm^−1^ is linked to C-H bending at C-1 of β galactopyranosyl [26,28]. The band assigned to the C-O vibration of 3,6-anhydro-galactose was detected at 928 cm^−1^ [29,30]. The intense band detected at 1033 cm^−1^ and the one at 1148 cm^−1^ are usually ascribed to C-O and C-C stretching vibrations of the pyranose ring [26,27]. The bands identified at 1243 cm^−1^ and 1367 cm^−1^ are assigned to the stretching vibration of ester sulfate groups [27,29,31]. The spectrum of extracted agar with alkaline pretreatment (Figure 2b) depicted an attenuation of the band at 1243 cm^−1^. It was previously reported that unstable sulfate could be removed during alkaline pretreatment [16].

#### 2.2.2. ^13^C-NMR Analysis

**Figure 3:**
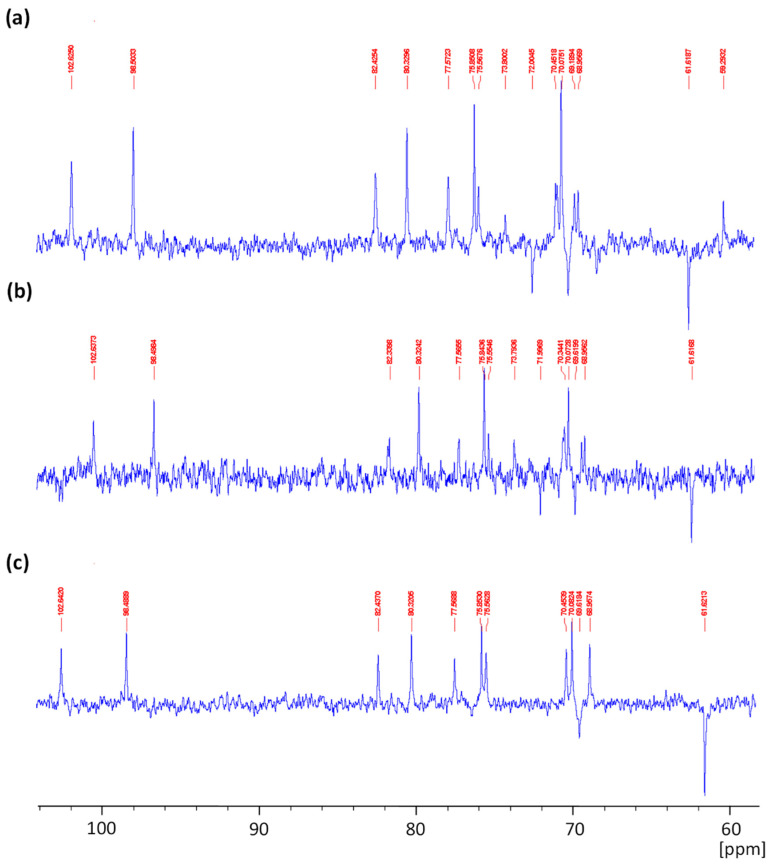
^13^C-NMR spectra of native (**a**) and alkaline pretreated agar (**b**) extracted from *Gracilaria gracilis* compared to commercial agar (**c**).

The ^13^C-NMR spectra of agar from *Gracilaria gracilis* (Figure 3a,b) are similar to that of commercial agar (Figure 3c), showing 12 signals characteristic of agarobiose. The signal detected at 102.6 ppm corresponds to C1 of β-d-galactopyranose, and those recorded at 70.4, 82.4, 68.9, 75.5, and 61.6 ppm are associated with C2, C3, C4, C5, and C6, respectively, of 3-linked β-d-galactopyranosyl units [32]. Furthermore, the signals for carbon atoms in 3,6-anhydro-α-l-galactopyranose were identified at 98.5, 80.3, 77.5, 75.8, 70.07, and 69.6 ppm related to C1, C3, C4, C5 C6, and C2, respectively [32,33,34]. The native extracted agar (Figure 3a) showed a weak signal at 59.2 ppm characteristic of O-methyl groups of agarobiose [32,35]. In contrast, signals linked to methoxy substituents were not noticeable in the spectrum of alkaline pretreated agar (Figure 3b). The minor signals detected in agar from *G. gracilis* at 72.0 and 73.8 ppm could be assigned to the residue of floridean starch [32,34].

### 2.3. Physical Properties of Agar Gels

#### 2.3.1. Gel Strength

The gel strength of agar extracted from *Gracilaria gracilis* varied from 105 ± 6.08 g·cm^−2^ for the native agar to 377 ± 19.79 g·cm^−2^ when 6% NaOH solution was used for pretreatment (Table 2). The comparison of gel strength values of agars from *G. gracilis*, previously reported in the literature, resulted in extreme variability. The gel strength of agar from *G. gracilis* from Thau lagoon (France) reached a value of 630 ± 15 g·cm^−2^ [20]. However, the gel strength value of *G. gracilis* from the Sea of Japan (Russia) did not exceed 250g·cm^−2^ [22]. Rebello et al. [36] reported a value of 859 g·cm^−2^ of agar gel strength for *G. gracilis* from Namibia. Likewise, the agar gel strength of *G. gracilis* from a Sicilian lagoon (Italy) showed a very high value of 880 g·cm^−2^ [37], while *G. gracilis* from the Patagonic coast of Argentina had a gel strength of 437 g·cm^−2^ [38]. This variability in gel strength values could be attributed to the different locations and physiological factors. Additionally, harvesting season is a determining factor for agar quality [39].

#### 2.3.2. Gelling and Melting Temperatures

Gelling and melting temperatures of the native extracted agar were 31.7 ± 0.2 °C and 78.5 ± 0.4 °C, respectively. As shown in Table 2, the alkaline-pretreated agar had high melting and gelling temperatures (82.1 ± 0.1 °C and 35.4 ± 0.3 °C, respectively). This result indicated that alkaline pretreatment contributed to improving the gelling and melting temperatures of agar from *G. gracilis*. The gelling (31.7–35.4 °C) and melting temperature (78.5–82.1 °C) ranges obtained for the *G. gracilis* agars are comparable to those reported by Rodríguez et al. [38] for the same species (31 °C and 85 °C, respectively). These values were lower than that reported for other *Gracilaria* species [18,21,40,41]. It has been reported that the gelling and melting temperatures were related to the harvested regions, extraction processes, and molecular weight distribution [16,21].

### 2.4. Chemical Properties

#### 2.4.1. Sulfate Content

**Figure 4:**
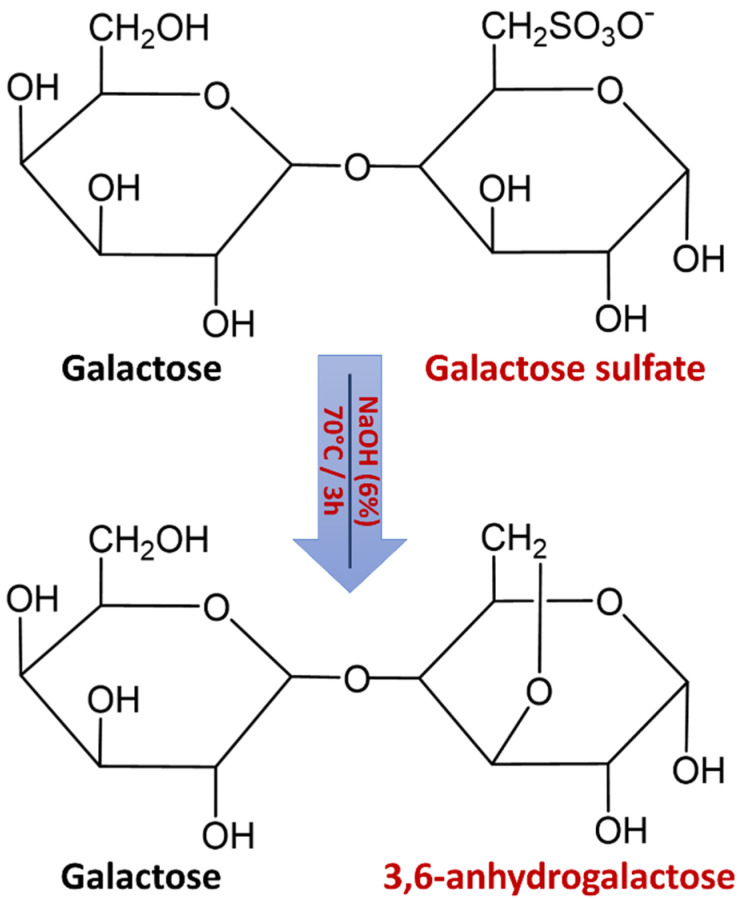
Schematic illustration of agar desulfation during alkaline treatment.

The extracted agars from *G. gracilis* showed low sulfate levels (Table 3). The native agar form of *G. gracilis* depicted relatively higher sulphate content (0.65 ± 0.03%) compared to that with alkaline pretreatment (0.32 ± 0.10%). Mollet et al. [24] reported that sulphate contents of *G. gracilis* from Roscoff (Brittany, France) were reduced to 2.1% after alkali treatments. The sulfate contents in *G. salicornia* were in the range of 0.3–0.8% of alkali-treated samples, but they exceeded 2% for native (non-treated) samples [42]. Moreover, the lowest sulfate content (1.8% ± 0.03%) of agar from *G. lemaneiformis* was detected for the alkaline treatment [43]. These results were attributed to the desulfation of alkaline treatment prior to agar extraction (Figure 4) that can eliminate the sulfate ester at C-6 of the l-galactose [44]. If the hydroxyl group on C-3 is free, the treatment of agar molecules with alkali solutions removes the energetically unstable axial sulphate ester at C-6 of the l-galactopyranose unit giving rise to more stable 3,6-anhydro-l-galactose [45]. This conversion led to the formation of a three-dimensional gel network, consequently improving the gelling properties [46].

#### 2.4.2. 3,6-Anhydro-galactose Content

As shown in Table 3, the content of 3,6-anhydro-galactose in *Gracilaria gracilis* agar was increased (11.85 ± 0.42%) when the algal biomass was treated with a NaOH solution. This observation supports the conclusion that the conversion of l-galactose sulfate to 3,6-anhydro-galactose by the alkaline treatment gave rise to the gel strength enhancement (Table 2). Similar findings were previously reported for other *Gracilaria* species [20,21,41,47]. Significant 3,6-anhydrogalactose contents are frequently related to high gel strength [9]. During the alkali pretreatment, the sulphate axially associated with 1,4-l-galactose is de-esterified resulting in an increase in 3,6-anhydro-galactose and amplification of the hydrogen bonds between extra hydroxyl groups and the oxygen atom linking the third and sixth carbon atoms in 1,4-l-galactose. Hence, the microcrystalline structure of agar becomes more stable [48].

## 3. Materials and Methods

The study site is located along the Dakhala shoreline (Southern Atlantic coast of Morocco). This area consists of a sheltered coastal bay where the largest zones of intertidal habitat are located in the northern part of the bay and along the inner coast of the peninsula. *Gracilaria gracilis* thalli were harvested from the outer coast of the peninsula (23°51′29″ N 15°51′58″ W) including a mixture of rocky and sandy biotope, with irregular-sized beaches and rocky outcrops. The collected samples were washed with sea water to remove the attached shells, sand, and other algae. Subsequently, the samples were thoroughly washed with tap water, and then subjected to sunlight for 1 week. The sundried samples were further dried to a constant weight at 50 °C.

The agar extraction was performed according to the methodology from Rebello et al. [49] and Li et al. [50], albeit slightly modified. Each extraction was performed in triplicate to obtain the average and standard deviation. Native agar was extracted by 20 g of dried seaweed hydrated in 500 mL of distilled water and heated at 100 °C for 2 h. The mixture was filtered, and the filtrate was allowed to gel at room temperature, frozen overnight, and thawed. The thawed gel was dehydrated with ethanol and then oven-dried at 50 °C to a constant weight. For the alkaline pretreatment extraction, 20 g of dried seaweed was mixed with 500 mL of a 6% NaOH solution and heated at 70 °C for 3 h. The residue was rinsed and soaked in 500 mL of distilled water at pH 6.2 for 12 h. The rest of the extraction steps proceeded as described in the native extraction.

The samples of agar powder were analyzed by FT-IR in attenuated total reflectance (ATR) mode using a Thermo Scientific Nicolet Impact 400D FT-IR Spectrometer (Nicolet Instrument Co., Madison, WI, USA). The spectra were scanned at a 4 cm^−1^ resolution between 500 and 4000 cm^−1^, with an average of 32 scans. The spectra were processed by OMNIC software (Nicolet, Madison, WI, USA).

The ^13^C NMR analyses of agar samples were carried out at 353 K using the spectrometer AV II 400 MHz, 9.4T (Proton Larmor frequency of 400.33 MHz, Bruker Corporation, Billerica, MA, USA), with a 5 mm Triple Resonance Broadband Inverse probe (Bruker Corporation, Billerica, MA, USA) at a base frequency of 100.62 MHz. Presaturation was applied during the relaxation delay and mixing time. The exponential multiplication apodization functions were applied in one dimension with 0.5 for line broadening prior to Fourier transformation.

The gel strength of the extracted agar (1.5% *w/v*) was investigated through determination of the load (g·cm^−2^), leading the cylindrical plunger (1 cm^2^ cross-section) to break the gel in 20 s [51]. Gelling and melting temperatures were measured according to the Freile-Pelegrın and Robledo [19] modified method. Agars (1.5% (*w/v*) were dissolved in distilled water at 90 °C. Then, 20 mL of agar solutions were poured in test tubes with glass beads (5 mm diameter). The tubes were regularly tilted until the bead stopped moving and the gel temperature was directly recorded. The melting temperature was tested using the same tubes by increasing the temperature from 50 to 100 °C at 0.5 °C/ min; as the bead dropped into the solution, the melting temperature was measured.

The sulphate content was measured by the BaCl_2_ turbidimetric slightly modified method of Craigie et al. [52]. Gelatin was dissolved in distillated water (0.3% *w/v*) at 60–70 °C and then allowed to cool. After 16 h at 4 °C, the temperature was brought to 20–25 °C and 2.0 g of BaCl_2_ was added to the gelatin solution. Then, 0.02 g of agar samples was hydrolyzed in 0.5 mL of HCl (2 N) for 2 h at 100 °C. The contents were then transferred and made to volume in a 10 mL volumetric flask. Humic substances were discarded by centrifugation. Furthermore, 1 mL of the supernatant, 9 mL of distilled water, and 1 mL of 0.5N HCl were mixed. Then, 0.5 mL of the BaCl_2_-gelatin reagent was added and agitated. After 30 min, the contents of the flask were again mixed, and the turbidity was measured at 550 nm against a reagent blank. The K_2_SO_4_ was used as a standard at the concentration range of 5–60 μg·S·mL^−1^.

The content of 3,6-anhydro-galactose was determined by the slightly modified resorcinol method [53,54]. The resorcinol reagent was prepared with 9 mL of a resorcinol solution (1.5 mg/mL), 1 mL of a 1,1-Diethoxyethane solution (0.04% *v/v*), and 100 mL of concentrated HCl. Subsequently, a 0.09 mL aliquot of the sample solution (1 mg/mL) was added to a glass tube followed by the addition of 0.6 mL of distilled water. After being placed in an ice bath for 5 min, 3 mL of the resorcinol reagent was added, mixed homogenously in an ice bath, and then kept at room temperature for 2 min. The mixture was incubated for 10 min at 80 °C followed by cooling for 5 min in an ice bath. The absorbance of 3,6-anhydro-galactose was measured at 555 nm. A standard curve was prepared using D-fructose at concentrations ranging from 1 to 40 μg·mL^−1^. The 3,6-anhydro-galactose content was calculated and expressed as the percentage (dry weight basis).

## 4. Conclusions

Agar extracted from the Rhodophyte *Gracilaria gracilis*, harvested from the Southern Moroccan Atlantic coast, was investigated via physical and chemical analysis. An agar yield of 20.5% ± 1.3% was obtained from alkali pretreated samples. The alkali pretreated agar demonstrated a better gelling property supported by higher gel strength (377 ± 19.79 g·cm^−2^), gelling (35.4 ± 0.3 °C), and melting (82.1 ± 0.1 °C) temperatures with a notable increase in 3,6-anhydro-galactose (11.85 ± 0.42%) and a decline in sulphate (0.32 ± 0.10%) contents. The agar from *G. gracilis* can ultimately be used in the agar industry once its physico-chemical properties have been enhanced by alkali treatment.

## Figures and Tables

**Table 1:** Agar yields of *Gracilaria gracilis*.

Extraction	Yield (% dw)
Native Extraction	15.16 ± 2.5
Alkaline Pretreatment	20.50 ± 1.3

**Table 2:** Gel proprieties of agar from *Gracilaria gracilis*.

Extraction	Gel Strength (g·cm^−2^)	Melting Temperature (°C)	Gelling Temperature (°C)
Native Extraction	105.30 ± 6.08	78.5 ± 0.4	31.7 ± 0.2
Alkaline Pretreatment	377.39 ± 19.79	82.1 ± 0.1	35.4 ± 0.3

**Table 3:** Sulphate and 3,6-anhydro-galactose contents of agar from *Gracilaria gracilis*.

Extraction	Sulfate (% dw)	3,6-AG (% dw)
Native Extraction	0.65 ± 0.03	5.67 ± 0.49
Alkaline Pretreatment	0.32 ± 0.10	11.85 ± 0.42

## Data Availability

All data are reported within this manuscript.
